# Would you test for 5000 Shillings? HIV risk and willingness to accept HIV testing in Tanzania

**DOI:** 10.1186/s13561-015-0060-8

**Published:** 2015-08-19

**Authors:** Jan Ostermann, Derek S. Brown, Axel Mühlbacher, Bernard Njau, Nathan Thielman

**Affiliations:** 1 Duke Global Health Institute, Duke University, Box 90392, 310 Trent Drive, Durham, NC 27701 USA; 2 Center for Health Policy and Inequalities Research, Duke University, Durham, NC USA; 3 Arnold School of Public Health, University of South Carolina, Columbia, SC USA; 4 Brown School, Washington University in St. Louis, St. Louis, MO USA; 5 Institut Gesundheitsökonomie und Medizinmanagement, Hochschule Neubrandenburg, Neubrandenburg, Germany; 6 Community Health Department, Kilimanjaro Christian Medical College, Moshi, Tanzania; 7 School of Medicine, Duke University, Durham, NC USA

**Keywords:** HIV testing, Tanzania, Sub-Saharan Africa, Willingness to accept, Contingent valuation, Incentives

## Abstract

**Objectives:**

Despite substantial public health efforts to increase HIV testing, testing rates have plateaued in many countries and rates of repeat testing for those with ongoing risk are low. To inform policies aimed at increasing uptake of HIV testing, we identified characteristics associated with individuals’ willingness-to-accept (WTA) an HIV test in a general population sample and among two high-risk populations in Moshi, Tanzania.

**Methods:**

In total, 721 individuals, including randomly selected community members (*N* = 402), female barworkers (*N* = 135), and male Kilimanjaro mountain porters (*N* = 184), were asked in a double-bounded contingent valuation format if they would test for HIV in exchange for 2000, 5000 or 10,000 Shillings (approximately $1.30, $3.20, and $6.40, respectively). The study was conducted between September 2012 and February 2013.

**Results:**

More than one quarter of participants (196; 27 %) stated they would be willing to test for Tanzania Shilling (TSH) 2000, whereas one in seven (98; 13.6 %) required more than TSH 10,000. The average WTA estimate was TSH 4564 (95 % Confidence Interval: TSH 4201 to 4927). Significant variation in WTA estimates by gender, HIV risk factors and other characteristics plausibly reflects variation in individuals’ valuations of benefits of and barriers to testing. WTA estimates were higher among males than females. Among males, WTA was nearly one-third lower for those who reported symptoms of HIV than those who did not. Among females, WTA estimates varied with respondents’ education, own and partners’ HIV testing history, and lifetime reports of transactional sex. For both genders, the most significant association was observed with respondents’ perception of the accuracy of the HIV test; those believing HIV tests to be completely accurate were willing to test for approximately one third less than their counterparts. The mean WTA estimates identified in this study suggest that within the study population, incentivized universal HIV testing could potentially identify undiagnosed HIV infections at an incentive cost of $150 per prevalent infection and $1400 per incident infection, with corresponding costs per quality adjusted life year (QALY) gained of $70 for prevalent and $620 for incident HIV infections.

**Conclusions:**

The results support the value of information about the accuracy of HIV testing, and suggest that relatively modest amounts of money may be sufficient to incentivize at-risk populations to test.

## Background

HIV counseling and testing (HCT) is a cost-effective means of primary and secondary HIV prevention and a point of entry into HIV care and treatment [1–3]. Despite substantial public health efforts to increase HIV testing, particularly in populations with generalized HIV epidemics, HIV testing rates have plateaued. In addition, rates of repeat testing among persons with ongoing risk are low. For example, in Tanzania, where there is widespread availability of varied HIV testing options, more than half of men and women ages 15–49 have never tested, and fewer than one-third reported testing in the previous 12 months [4]. Testing rates remain low even among populations at extreme risk: a recent report by the Joint United Nations Programme on HIV/AIDS described that across 35 sub-Saharan African (SSA) countries, only 60 % of female sex workers had received an HIV test result in the past 12 months [5].

Basic economic theory states that rational, forward-looking individuals will test for HIV if the expected (cumulative) benefits are greater than the expected (cumulative) costs, including disutility and unpleasantness of the test itself, the discomfort associated with receiving potentially negative information, and the opportunity costs of testing (e.g., transportation costs, lost household or labor market productivity, etc.) [6]. This basic framework applies in resource-rich as well as resource-poor settings such as SSA, where decades of health promotion, education, and medical advances have significantly altered the landscape of HIV prevention, diagnosis and treatment [7].

HIV testing provides several potential benefits: a positive test result establishes the diagnosis of HIV infection, which is the first step for accessing effective, life-prolonging treatment for an otherwise fatal disease, whereas an HIV-negative test result removes uncertainty about the individual’s serostatus. However, there may be offsets to these benefits, such as the expected cost of having to live with the information from a positive test result (e.g., stress, social isolation, guilt) [8, 9]. Like several other diagnostic tests with similar benefits, the behavior of a person who actually tests for HIV reveals an implicit value of the test (implicit willingness to pay, WTP), which in some cases corresponds to a substantial amount of money [10, 11].

However, among populations with limited resources, particularly among most parts of SSA, the value of HIV testing to an individual may not exceed the opportunity costs (e.g., food, housing), and behavioral economics suggests that individuals may not always act rationally according to classic economic theory [12, 13]. In the only WTP study in SSA (conducted among persons who were not actually testing), half were unwilling to pay any amount of money for an HIV test [14]. Further, despite the widespread availability of free HIV testing in SSA, and significant increases in the number of clients presenting for free vs. fee-based HCT [2], substantial numbers of people do not test. These results suggest that, consistent with economic theory on other non-financial costs from testing, financial costs are not the only barrier, and that individuals’ valuations of other costs, including the time and monetary cost of accessing testing venues, fears of knowing the result, and HIV-related stigma, outweigh their valuations of the expected benefits of a test [6].

In theory, subsidies in the form of incentives can change an individual’s cost structure, in that incentives offset financial and non-financial costs and “tip the scale” toward making HIV testing and repeat testing a utility maximizing choice in a simple benefit-cost (or more sophisticated forward-looking) model [15–17]. Incentives may thus be used to align individual and social preferences for specific behaviors [18]. Financial and non-financial incentives have been used to encourage the use of diverse preventive and health behaviors [19–23], including to promote HIV risk reduction [24–29]. However, research on the potential effectiveness of incentives to encourage HIV testing has been limited. A recent review [30] identified only 3 studies that evaluated the effect of incentives for HIV testing – one in the United States [31] and two in sub-Saharan Africa [32, 33]; incentives were associated with increased rates of clients completing HCT [31, 33] and with increased testing uptake among higher-risk populations [32].

Importantly, none of the studies assessed the potential incentive range required to increase testing, nor did these studies characterize variation in the balance between benefits and costs (both direct financial costs and non-financial barriers to testing) across individuals. In theory, an incentive only needs to be as large as the barrier [34], thus, the optimal incentive amount varies with individuals’ perceived benefits and barriers to testing. To inform policies aimed at increasing uptake of HIV testing, we analyzed variation in the net value that individuals place on barriers to testing. Specifically, we identify characteristics associated with individuals’ willingness-to-accept (WTA) an HIV test in a general population sample and in two high-risk populations using a double-bounded contingent valuation format.

## Methods

### Setting

The study was conducted between September 2012 and February 2013 in Moshi, Tanzania. In 2012, the town had a population of 184,292 [35], with an estimated HIV prevalence of 3.8 % [4]. Public health officials in the study area identified two populations at particularly high risk of HIV infection: female barworkers and male mountain porters. HIV prevalence among barworkers in this area has been estimated at 19 to 26 % [36, 37]. Kilimanjaro mountain porters [38] are predominantly young males, face volatile income cycles, and spend extended time away from home, thus sharing many characteristics with other high-risk groups, such as long-distance truck drivers [39, 40], fishermen [41–43], miners [44, 45], and migrant farm workers [46]. HCT services in the study area are available, free of charge, at hospitals, health centers, and free-standing voluntary counseling and testing facilities. Intermittently, mobile and outreach testing options have also been available at venues such as schools, markets, or workplaces. For clients who test HIV positive, 8 HIV care and treatment centers provide access to antiretroviral therapy [47].

### Sample

The characteristics and HIV testing preferences of 486 randomly selected community residents and two high-risk populations participating in the *HIV Testing Preferences in Tanzania* study (2012–2014) were previously described [48, 49]. In short, cluster-randomization and *Expanded Programme on Immunization* sampling methodology [50] were used to enroll a random sample of male and female community members from an urban setting in Northern Tanzania. Forty *mitaa* (singular: *mtaa*, an administrative area translated as ‘neighborhood’) within Moshi Municipality were randomly selected. Within each *mtaa*, a randomly selected GPS coordinate was used to identify a house that served as the starting point for participant enrollment. In each household, one adult age 18–49 was randomly selected for an in-person interview in the respondent’s home. Snowball sampling was subsequently used to recruit 162 female barworkers and 194 male mountain porters of the same age range in the same area. Seed participants were recruited from barworkers presenting for a health check-up at a municipal health center and from climbing companies and a porters union. Eligible persons were invited to a research office and verbally consented into the study. Participants who reported to have never been sexually active (*N* = 87) and those who reported to have previously tested positive for HIV (*N* = 34) were excluded from analyses for this manuscript.

### Survey

Surveys were administered by trained interviewers in participants’ native language, Kiswahili, to assess HIV testing history and plans, preferences for various actual or hypothetical HIV testing options, as well as sociodemographic and HIV risk characteristics. Protocols for key aspects of the research implementation and low refusal rates (below 5 %) have been previously described [48].

The survey included dichotomous choice questions that assessed participants’ willingness to test for HIV in exchange for a monetary payment. Prior qualitative work [6] and pre-tests of the WTA questions suggested a mistrust of payments for HIV testing; therefore, WTA questions were prefaced with an introductory script: “*You said it takes time to test*, *and testing may interfere with your normal activities. If someone asked you to change your plans for tomorrow in order to test for HIV*, *it may be appropriate if they compensate you for your time*.”

Following the introductory script, participants were asked: “*If you were offered 5000 Shilling to test for HIV tomorrow, would you test?*” Those who responded positively were asked “*If you were offered 2,000 Shilling to test for HIV tomorrow, would you test?*”; those who responded negatively to the first question were asked “*If you were offered 10,000 Shilling to test for HIV tomorrow, would you test?*”. As a result, the dichotomous choice questions resulted in 4 possible outcomes:

- “Yes” to the offer of 5000 and again “yes” to the offer of 2000
- “Yes” to the offer of 5000 and “no” to the offer of 2000
- “No” to the offer of 5000 and “yes” to the offer of 10,000
- “No” to the offer of 5000 and again “no” to the offer of 10,000

Participants who were not willing to test for the highest bid amount were asked a follow-up question: “*How much would they have to offer so that you would test for HIV tomorrow?*” The incentive range was selected based on focus groups and pretests, in which the salient range from low to high acceptability among the sample was identified. At the time of the study 1 US Dollar was worth approximately 1,550 TSH. The cutoff values of 2,000 TSH, 5,000 TSH and 10,000 TSH corresponded to approximately $1.30, $3.20, and $6.40, respectively. The average gross domestic product (GDP) per capita at the time of the study was approximately $835 per year, or $2.30 per day.

### Model

We assume that individuals maximize utility and face a tradeoff between testing and the level of consumption of all other goods, subject to resource constraints in terms of income (unobserved on this survey) and time (an identical endowment for all individuals). The societal perspective, promoted by public health officials and clinicians, is that the value of information is unambiguously positive because it may change health-related behaviors, including linkage to care for those found to be HIV positive, and improve others’ welfare through reduced HIV transmission risk. From the individual’s perspective, however, the private value of information is ambiguous, depending on preferences, risks, and individual characteristics. Clearly, revealed preferences in the form of low observed testing rates indicate that for many persons testing is not utility-maximizing in equilibrium. The time and travel costs of seeking testing are negative even if there is generally no financial cost to testing in our study area*.* An individual demands HIV testing—or not testing—by considering the benefits and costs of the test. Potential compensation for HIV testing (WTA) may alter the demand for testing, and private benefits and costs are captured through several covariates in the survey.

The value of information is a function of an individual’s probability of being infected with HIV: a higher probability of infection corresponds to a greater likelihood that the test results in access to effective, life-prolonging medications. The risk of HIV infection is captured in our data by the participant’s age in years (*a*), the number of lifetime sexual partners (*s:* 1*–*2, 3–4, or 5+), the presence of HIV-related symptoms (*h:* any fever, cough, coughing up blood, sweating at night, diarrhea, genital ulcers, or rash) in the past 3 months, and reports of commercial sex (*c*), defined as having ever given or received gifts or money for sex.

The value of information also depends on the perceived accuracy of the HIV test (*pa*), which was assessed by the question: “*In your opinion, how accurate are HIV tests?*”, with answer options: “*inaccurate*”, “*mostly accurate*”, and “*completely accurate*”. An indicator variable for “completely accurate” was included in the final model. Preferences for testing and the value of information may depend on experience with HIV testing, therefore an indicator variable for those who previously tested for HIV (*pt*) was also included as a covariate.

HIV testing is freely available in diverse settings in the study area; therefore the cost of testing primarily consists of the opportunity cost of time. To approximate opportunity cost and time preferences, we included participants’ education (*e:* any secondary education vs. primary education or less) as a covariate. Education may also be considered a marker of health literacy [51]. The occupation-based selection of the two high-risk groups precluded the use of an occupation- or employment-based measure of opportunity cost.

Marital status (*m*) and a sexual partner’s testing history (*st*) may affect both the benefits and costs of testing and were included as indicators of both risk and barriers. Married individuals may be less likely to have multiple concurrent sexual partners (i.e., lower risk); they may be more concerned about the implications of a positive test result (i.e., greater barriers); and they may feel greater responsibility to others (i.e., greater benefit). A partner having tested for HIV may reduce barriers to testing, particularly for females, but may also change an individual’s perception of risk, as some consider their spouse’s HIV test a proxy test [52].

Combining these factors, the demand for testing is a function of the “price” of testing (*q*), the price of all other goods (*p*), a resource endowment (*y*), and random factors (*ε*) representing unmeasured preferences or measurement error. The individual utility function is expressed as *V*(*p, q, y, ε*). The price of testing is a function of the characteristics discussed above, *q = f(a, e, h, s, c, pt, m, st, pa*). *A priori,* the demand for testing, *q* ^*0*^, represents a utility-maximizing choice weighing the consumption of testing (a function of perceived benefits and costs of testing) against the consumption of other goods. On our survey, the WTA questions alter the hypothetical utility function, and demand for testing may change from *q* ^*0*^ to *q* ^*1*^. Testing will decrease private utility for all who did not previously seek it, or *V*(*p, q* ^*1*^ *, y, ε*) < *V*(*p, q* ^*0*^ *, y, ε*), unless offset through compensation, *WTA*, which enables the purchase of other goods:

$$ V\left(p,\kern0.5em {q}^0,\kern0.5em y,\kern0.5em \varepsilon \right)=V\left(p,\kern0.5em {q}^1,\kern0.5em y+WTA,\kern0.5em \varepsilon \right) $$

The prices of other goods (*p*) are constant in both scenarios and cancel out of the econometric model, so they are not included in the model or available on our survey. Income (*y*) is also not available, although it is captured indirectly through education.

For cultural [53, 54] and epidemiological [55, 56] reasons, HIV risk and willingness to accept an HIV test were expected to differ by gender; therefore gender-specific models were estimated. Among females and among men, generalized Hausman tests [57] indicated that parameter estimates did not differ significantly between randomly selected community members and the respective high-risk population (female barworkers: chi-square (10df) =9.41; *p* = 0.4935; male mountain porters: chi-square (10df) =5.02; *p* = 0.8895), thus the community sample and high-risk groups were pooled within each gender.

### Estimation

Because amounts were fixed across respondents, the probabilities of observing the four possible response patterns to the two questions are described as follows:

$$ \begin{array}{l}{\pi}^{y,y}= \Pr \left( \min \kern0.5em WTA\kern0.5em \le \kern0.5em \mathrm{T}\mathrm{S}\mathrm{H}\kern0.5em 2000\right)\hfill \\ {}{\pi}^{y,n}= \Pr \left(\mathrm{T}\mathrm{S}\mathrm{H}\kern0.5em 2000< \min \kern0.5em WTA\kern0.5em \le \kern0.5em \mathrm{T}\mathrm{S}\mathrm{H}\kern0.5em 5000\right)\hfill \\ {}{\pi}^{n,y}= \Pr \left(\mathrm{T}\mathrm{S}\mathrm{H}\kern0.5em 5000< \min \kern0.5em WTA\kern0.5em \le \kern0.5em \mathrm{T}\mathrm{S}\mathrm{H}\kern0.5em 10000\right)\hfill \\ {}{\pi}^{n,n}= \Pr \left( \min \kern0.5em WTA>\mathrm{T}\mathrm{S}\mathrm{H}\kern0.5em 10000\right)\hfill \end{array} $$

Or more generally, let these probabilities be indicated by π^c1, c2^, where *c*_*t*_ is the choice (*y* = yes, *n* = no) to bid *t* (1 = first, 2 = second). To estimate WTA, we assume a normally distributed error term that is uncorrelated across individuals. The distribution of observed responses is given by the following function:

$$ \begin{array}{l}{\displaystyle \sum_{i=l}^N\left[{d}_i^{y,y} \ln \left(\Phi \left({z}_i^{\hbox{'}}\frac{\beta }{\sigma }-\frac{-2000}{\sigma}\right)\right)+{d}_i^{y,n} \ln \left(\Phi \left({z}_i^{\hbox{'}}\frac{\beta }{\sigma }-\frac{-5000}{\sigma}\right)-\Phi \left({z}_i^{\hbox{'}}\frac{\beta }{\sigma }-\frac{-2000}{\sigma}\right)\right)\right.}\hfill \\ {}+{d}_i^{n,y} \ln \left(\Phi \left({z}_i^{\hbox{'}}\frac{\beta }{\sigma }-\frac{-10000}{\sigma}\right)-\Phi \left({z}_i^{\hbox{'}}\frac{\beta }{\sigma }-\frac{5000}{\sigma}\right)\right)+{d}_i^{n,n} \ln \left(1\right.\hfill \\ {}\left.\left.-\Phi \left({z}_i^{\hbox{'}}\frac{\beta }{\sigma }-\frac{-10000}{\sigma}\right)\right)\right]\hfill \end{array} $$

where the individuals’ binary choices are represented by the mutually exclusive indicator variables *d* ^*y*,*y*^, *d* ^*y*,*n*^, *d* ^*n*,*y*^, *d* ^*n*,*n*^ as in [58]. Parameters were estimated using maximum likelihood with the Stata *doubleb* routine [58] for double-bounded dichotomous choice contingent valuation models [59]. Because study questions here elicited willingness-to-accept rather than willingness-to-pay, the estimated coefficients were multiplied with −1. Models were estimated in Stata v.13.1.

### Ethics statement

Study activities were approved by the Institutional Review Boards of Duke University and Kilimanjaro Christian Medical University College, and Tanzania’s National Institute for Medical Research. Participants provided written informed consent and received an incentive of 3,000 Tanzania Shilling (TSH) for participation in the study.

## Results

### Sample characteristics

Sample characteristics, by gender and risk group, are shown in Table 1. On average, participants were just under 30 years old, with one-third of women and half of men reporting any secondary education. Half of women and 40 % of men reported symptoms of HIV in the past 3 months. High risk of HIV infection is indicated by one-quarter of women and one-half of men reporting 5 or more lifetime sexual partners; commercial sex was reported by 61 % of female participants and 73 % of male participants. Ninety percent of women and two-thirds of men had previously tested for HIV, with half of respondents reporting that their partner had tested. Approximately one-third of participants perceived HIV tests to be less than “completely accurate.”

**Table 1:** Basic demographic and risk characteristics of participants (N=721)

		Females					Males				
		Community		Barworkers			Community		Porters		
Variable	Value	Mean (sd) or N (%)		Mean (sd) or %			Mean (sd) or %		Mean (sd) or %		
Age	Years	29.8	(7.7)	27.7	(5.4)	^a^	26.6	(7.5)	29.5	(6.9)	^b^
Education	Primary education or less	193	(67.2 %)	86	(63.7 %)		43	(37.4 %)	105	(57.1 %)	^b^
	Secondary education	94	(32.8 %)	49	(36.3 %)		72	(62.6 %)	79	(42.9 %)	
Symptoms of HIV, past 3 months	No symptoms	155	(54.0 %)	48	(35.6 %)	^b^	72	(62.6 %)	108	(58.7 %)	
	Any symptoms	132	(46.0 %)	87	(64.4 %)		43	(37.4 %)	76	(41.3 %)	
# of lifetime sexual partners	1-2	189	(65.9 %)	8	(5.9 %)	^b^	49	(42.6 %)	24	(13.0 %)	^b^
	3-4	80	(27.9 %)	32	(23.7 %)		39	(33.9 %)	49	(26.6 %)	
	5+	18	(6.3 %)	95	(70.4 %)		27	(23.5 %)	111	(60.3 %)	
Commercial sex	Never	155	(54.0 %)	9	(6.7 %)	^b^	60	(52.2 %)	22	(12.0 %)	^b^
	Ever	132	(46.0 %)	126	(93.3 %)		55	(47.8 %)	162	(88.0 %)	
Previously tested for HIV	Never	24	(8.4 %)	17	(12.6 %)		39	(33.9 %)	56	(30.4 %)	
	At least once	263	(91.6 %)	118	(87.4 %)		76	(66.1 %)	128	(69.6 %)	
Marital status	Not married	94	(32.8 %)	95	(70.4 %)	^b^	82	(71.3 %)	92	(50.0 %)	^b^
	Married	193	(67.2 %)	40	(29.6 %)		33	(28.7 %)	92	(50.0 %)	
Partner has tested for HIV	Partner has not tested	119	(41.5 %)	77	(57.0 %)	^a^	51	(44.3 %)	91	(49.5 %)	
	Partner has tested	168	(58.5 %)	58	(43.0 %)		64	(55.7 %)	93	(50.5 %)	
Perceived accuracy of the HIV test	Mostly accurate or inaccurate	81	(28.2 %)	40	(29.6 %)		42	(36.5 %)	55	(29.9 %)	
	Completely accurate	206	(71.8 %)	95	(70.4 %)		73	(63.5 %)	129	(70.1 %)	
N		287		135			115		184		

The significance of differences between high-risk populations and the respective general community samples was assessed using Student’s *t*-test, chi-squared test, or Fisher’s exact test, as appropriate

^a^, and ^b^ indicate statistical significance at the 0.01, and 0.001 levels, respectively

There were significant differences in participant characteristics by gender, and between randomly selected community members and high-risk groups (Table 1). Compared with females, males reported higher education (*p*<0.001), fewer potential HIV-related symptoms (*p*<0.01), and more lifetime sexual partners (*p*<0.001); they were less likely to have previously tested for HIV (*p*<0.001) and less likely to be married (*p*<0.001).

Compared with randomly selected female community members, female barworkers were younger (*p*=0.005), reported more HIV-related symptoms (*p*<0.001), more lifetime sexual partners (*p*<0.001), higher rates of commercial sex (*p*<0.001), and were less likely to be married (*p*<0.001) or have a partner who tested for HIV (*p*=0.003). Porters were older (*p*<0.001) and less educated (*p*<0.001), reported more lifetime sexual partners (*p*<0.001) and higher rates of commercial sex (*p*=0.001), and were less likely to be married (*p*<0.001), compared with randomly selected male community members.

### Willingness to accept an HIV test

**Fig. 1:**
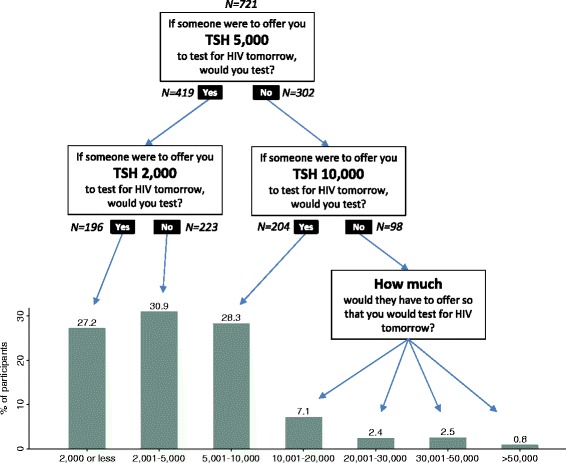
Distribution of WTA responses (2012/13 Tanzania Shillings; N=721). 6 of 98 respondents (0.8 % of the total sample) did not provide a numeric answer to the follow-up question of how much they would have to be offered to test for HIV tomorrow. Their binary choices (No; No) are included in the WTA analyses. At the time of the study 1 US Dollar was worth approximately 1,550 TSH. The cutoff values of 2,000 TSH, 5,000 TSH and 10,000 TSH correspond to approximately $1.30, $3.20, and $6.40, respectively

The distribution of responses to the WTA questions is shown in Fig. 1. Of 721 participants, 196 (27 %) stated that they would be willing to test for TSH 2,000 (or less), whereas 98 (13.6 %) said that they would require more than TSH 10,000. The latter group, in an open-ended follow-up question, was asked about the amount at which they would test; the highest amount was TSH 150,000 (~$100). More than half of participants (419 of 721 participants; 58.1 %) indicated that they would test for HIV if offered TSH 5,000; the number increased to 623 (86.4 %) in response to an offer of TSH 10,000.

**Fig. 2:**
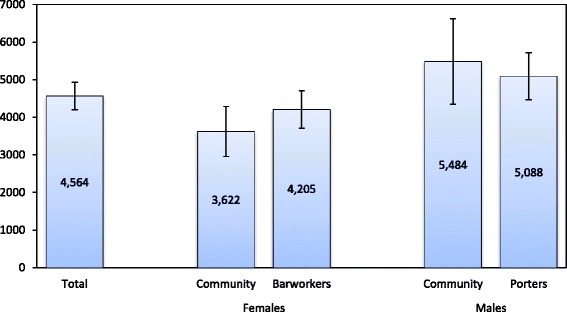
Mean WTA estimates from univariate double-bounded choice models, by gender and HIV risk group (2012/13; Tanzania Shillings)

The average WTA estimate was TSH 4,564 (US$ 2.94; 95 % Confidence Interval; CI: TSH 4201 to 4927; Fig. 2). Randomly selected male community members had the highest WTA values (US$ 3.54; TSH 5,484; 95 % CI: TSH 4,351 to 6,618), whereas female community members had the lowest values (US$ 2.34; TSH 3,622; 95 % CI: TSH 2,956 to 4,287).

The estimated WTA for an HIV test varied significantly by the expected benefit and cost measures included in our model, namely respondents’ risk characteristics and opportunity cost (Table 2). Among females, secondary education (*p*<0.01) and a previous HIV test (*p*<0.05) were associated with higher WTA values, whereas females who reported to have engaged in commercial sex and those whose partner had tested for HIV indicated lower WTA values (both *p*<0.05). Among males, HIV-related symptoms in the past 3 months were associated with a lower WTA (*p*<0.01). For both genders, the most significant association was observed with respondents’ perception of the accuracy of the HIV test, with those who believed that HIV tests are completely accurate indicating that they would test for approximately one third less (*p*<0.01).

**Table 2:** Marginal WTA estimates from gender-specific multivariate double-bounded choice models (2012/13 Tanzania Shillings)

	Females (N=422)			Males (N=299)		
	Coefficient	Std.err.	p-value	Coefficient	Std.err.	p-value
Age	−4.6	(34.1)	0.893	16.1	(55.5)	0.771
Any secondary education	1398.9 ^b^	(499.2)	0.005	1149.1	(618.9)	0.063
Any HIV symptoms	−722.0	(466.2)	0.122	−1654.9 ^b^	(570.9)	0.004
3-4 lifetime sexual partners	−208.0	(605.5)	0.731	1411.4	(791.9)	0.075
5+ lifetime sexual partners	593.5	(664.4)	0.372	365.4	(809.1)	0.652
Ever exchanged sex for gifts or money	−1273.3 ^a^	(564.2)	0.024	−172.2	(698.4)	0.805
Previously tested for HIV	1920.9 ^a^	(851.4)	0.024	−583.3	(651.7)	0.371
Married	−413.0	(530.9)	0.437	256.1	(853.3)	0.764
Partner tested	−987.7 ^a^	(501.4)	0.049	216.7	(703.6)	0.758
Test is perceived to be completely accurate	−1445.3 ^b^	(508.5)	0.005	−1820.3 ^b^	(588.9)	0.002

^a^ and ^b^ indicate statistical significance at the 0.05 and 0.01 levels, respectively

## Discussion

To our knowledge, this is the first study to use stated preference surveys to probe the range of economic incentives required for a pay-for-testing strategy in low-resource settings. Our study included both representative community samples and high-risk populations from each gender. We demonstrate that (1) relatively modest economic incentives are potentially useful in increasing HIV testing rates in this population, and (2) willingness to accept differs significantly by gender, with indicators of HIV risk, and with participants’ perceptions of the accuracy of the HIV test.

The results have important policy implications. First, financial incentives for HIV testing may be highly cost effective. HIV prevalence estimates of 3.8 % [60] and Census data [35] suggest that, of the 94,530 Moshi residents aged 18–49, approximately 3,600 live with HIV. Assuming, optimistically, that half of infected adults are already aware of their HIV infection [61], universal testing could plausibly identify 1,800 undiagnosed HIV cases in Moshi alone. Further, if national incidence estimates (0.32 % [62]) were rescaled to account for lower-than-average HIV prevalence in the study area (national HIV prevalence is estimated at 5.1 % [60]), universal annual repeat testing would be expected to identify about 200 new HIV infections each year. The mean WTA estimates identified in this study suggest that in this population universal testing could potentially be achieved with incentive costs of about $280,000, amounting to $150 per diagnosis of prevalent HIV infection, and $1400 per diagnosis of annual incident infections. With an estimated 2.25 discounted quality-adjusted life years (QALY) gained for each newly infected person identified [63], the incentive costs per QALY range from under $70 for prevalent HIV infections to $620 for incident infections. Additional QALYs would be gained from reduced numbers of secondary infections [63]. These estimates compare favorably to Tanzania’s 2012 per capita GDP of $835 [64] and to prior estimates of the cost effectiveness of regular HIV re-testing among high prevalence populations in low-resource settings [63].

Second, while many diverse characteristics influence an individual’s cost structure and thus their individual WTA, systematic variation in WTA by gender, risk and other characteristics plausibly reflects systematic variation in individuals’ valuations of benefits and barriers, and can thus inform the design of HIV testing interventions that target specific risk groups or specific barriers to testing. Larger WTA estimates, analogous to higher incentive amounts required to entice individuals to test, were observed for females who had completed secondary education. This observation is consistent with different time preferences as well as greater income potential and thus higher opportunity cost for secondary school graduates. An offer of HIV testing that provides expanded testing hours, reduced waiting time, or allows for appointments for testing, may appeal to those with the greatest opportunity cost.

Among females, a prior HIV test was also associated with higher incentive amounts. This observation is consistent with lower expected benefits from a second HIV test, as the likelihood of incident HIV infection since the last test is generally lower than the cumulative, lifetime risk of infection at the first test. A similar effect among males was not statistically significant at conventional levels, plausibly due to the smaller sample size. Policy messages aimed at increasing rates of repeat testing may be more effective if recommended repeat testing intervals take into consideration variation in individuals’ ongoing risk of HIV infection [63].

Indicators of HIV risk were associated with lower WTA estimates, an observation consistent with greater expected benefits from an HIV test. Females who had ever engaged in commercial sex required a smaller incentive amount than those who had not. The most plausible reason for this is that commercial sex workers are aware of their increased risk of HIV infection and perceive greater utility of testing. Alternatively, it is possible that commercial sex workers are more open to accepting incentives in exchange for certain behaviors (i.e. sex and HIV testing). Similarly, among males, lower required incentive amounts were observed among those with recent symptoms potentially related to HIV. Others have described high rates of late, (i.e. symptomatic) presentation of HIV disease among males seeking HIV testing [65]. Efforts should be expanded to incentivize males to test for HIV prior to the onset of symptoms.

Finally, the strong association of WTA with participants’ perception of test accuracy points to the potential value of information campaigns to correct misperceptions about the accuracy of HIV testing [66]. Perceived unreliability of test results and distrust of HIV testing technologies has been previously shown to discourage uptake of HIV testing [67–69]; our finding is consistent with an increased incentive amount required to encourage those to test who are less confident in the accuracy of the test results.

As the parameters for incentivized HIV testing are explored, we acknowledge several important ethical considerations that derive from conditional cash transfers for health interventions. These have been explored in more detail by others [17, 70–74]. From a policy perspective, some argue that in highly resource-constrained settings limited health care funds should be allocated to overcome supply-side obstacles, such as expanding health system capacity, before investing in cash incentives [75]. Others point to the potential for unintended consequences of cash transfer programs. Stecklov et al., for example, described an increase in fertility rates in a Honduran program that provided cash transfers targeting health, nutritional, and educational outcomes for children [76]; and evidence from Malawi suggests that financial incentives may have the perverse effect of increasing HIV risk behaviors as a result of additional disposable income [77]. The equitability of cash-incentivized health-interventions across economic strata and by gender is another potential concern. Moreover, establishing financial incentives for certain health behaviors, such as HIV testing, could set expectations that other health behaviors (e.g. attending antenatal clinic visits) should also be incentivized, which might indirectly result in decreased participation. Finally, concerns about the real-world implementation and long-term sustainability of incentives must be considered: There is evidence that extrinsic motivators, such as financial incentives, may crowd out intrinsic motivators [78], potentially reducing testing rates after incentives are no longer provided. These ethical considerations at the population level merit careful deliberation. On the individual level, the greatest ethical concern is the potential for coercion – namely undermining the recipient’s autonomy and the integrity of her/his own decision making process, with undue inducement [74]. If an individual is already predisposed to test for HIV and the incentive represents a gentle ‘nudge’ from inaction to action along a pathway in keeping with his/her considered values, we see no concerns. On the other hand, if the financial reward is so great relative to potential risks (e.g. potential spousal abuse for testing), then the incentive may constitute wrongful interference and undue coercion. Extensive formative research, careful piloting and ongoing modifications of cash incentives are necessary to guard against perverse incentives, unintended consequences, and the potential for coercion.

### Limitations

This study is subject to several limitations. First, starting bids were not randomized due to the survey’s administration as a paper survey. Different WTA estimates may have been obtained if starting values had been randomized [79, 80] or if the incentive structure had involved either losses or lotteries [81]. Second, the contingent valuation questions were part of a broader study, and we did not power the data collection specifically for the analyses described in this paper. Small sample sizes, particularly for males in the community sample, may have contributed to the lack of statistical significance of parameters describing opportunity cost (any secondary education) among males, and risk (number of sexual partners) in both genders. Third, the hypothetical nature of stated preference contingent valuation data may not reflect ‘real-world’ behaviors: WTA estimates based on stated preferences may differ from estimates that would be obtained on the basis of revealed preferences data [82, 83], and barriers specific to HIV, such as fear and stigma, may limit the acceptability of financial incentives in the context of HIV testing [6]. Fourth, further research is needed to evaluate the extent to which specific WTA estimates are transferable to other risk groups within and beyond the study area.

## Conclusion

This study used stated preference survey methods to probe the value of economic incentives required for a pay-for-testing strategy in a low-resource setting. The results support the value of information campaigns to correct misperceptions about the accuracy of HIV testing, and that relatively modest amounts of money may be sufficient to incentivize at-risk populations to test for HIV. For translation of the results to policy, additional implementation research will be required.

## Abbreviations

CI: Confidence interval
GDP: Gross domestic product
GPS: Global positioning system
HCT: HIV counseling and testing
HIV: Human immunodeficiency virus
QALY: Quality-adjusted life years
SSA: Sub-Saharan Africa
TSH: Tanzania Shilling
WTA: Willingness to accept
WTP: Willingness to pay

## References

[1] Sweat M, Gregorich S, Sangiwa G, Furlonge C, Balmer D, Kamenga C (2000). Cost-effectiveness of voluntary HIV-1 counselling and testing in reducing sexual transmission of HIV-1 in Kenya and Tanzania. Lancet.

[2] Thielman NM, Chu HY, Ostermann J, Itemba DK, Mgonja A, Mtweve S (2006). Cost-effectiveness of free HIV voluntary counseling and testing through a community-based AIDS service organization in Northern Tanzania. Am J Public Health.

[3] Dieffenbach CW, Fauci AS (2009). Universal voluntary testing and treatment for prevention of HIV transmission. JAMA.

[4] Tanzania Commission for AIDS. Tanzania HIV/AIDS and Malaria Indicator Survey 2011–2012. In*.*: TACAIDS, ZAC, NBS, OCGS, and ICF International; 2013.

[5] UNAIDS (2014). The GAP report.

[6] Njau B, Ostermann J, Brown D, Muhlbacher A, Reddy E, Thielman N (2014). HIV testing preferences in Tanzania: a qualitative exploration of the importance of confidentiality, accessibility, and quality of service. BMC Public Health.

[7] Merson MH, Curran JW, Griffith CH, Ragunanthan B (2012). The President's emergency plan for AIDS relief: from successes of the emergency response to challenges of sustainable action. Health Aff.

[8] Payne K, McAllister M, Davies LM (2013). Valuing the economic benefits of complex interventions: when maximising health is not sufficient. Health Econ.

[9] Grosse SD, Wordsworth S, Payne K (2008). Economic methods for valuing the outcomes of genetic testing: beyond cost-effectiveness analysis. Genet Med.

[10] Lin PJ, Cangelosi MJ, Lee DW, Neumann PJ (2013). Willingness to pay for diagnostic technologies: a review of the contingent valuation literature. Value Health.

[11] Forsythe S, Arthur G, Ngatia G, Mutemi R, Odhiambo J, Gilks C (2002). Assessing the cost and willingness to pay for voluntary HIV counselling and testing in Kenya. Health Policy Plan.

[12] Kahnemann D (2003). Maps of bounded rationality: psychology for behavioral economics. Am Econ Rev.

[13] The World Bank. World Development Report: Mind, Society, and Behavior. In*.* Washington, DC; 2015.

[14] Uzochukwu B, Uguru N, Ezeoke U, Onwujekwe O, Sibeudu T (2011). Voluntary counseling and testing (VCT) for HIV/AIDS: a study of the knowledge, awareness and willingness to pay for VCT among students in tertiary institutions in Enugu State Nigeria. Health Policy.

[15] Madrian BC (2014). Applying insights from behavioral economics to policy design. Annu Rev Econom.

[16] Rice T (2013). The behavioral economics of health and health care. Annu Rev Public Health.

[17] Heise L, Lutz B, Ranganathan M, Watts C (2013). Cash transfers for HIV prevention: considering their potential. J Int AIDS Soc.

[18] Bowles S, Polania-Reyes S (2012). Economic incentives and social preferences: substitutes or complements?. J Econom Lit.

[19] Ranganathan M, Lagarde M (2012). Promoting healthy behaviours and improving health outcomes in low and middle income countries: a review of the impact of conditional cash transfer programmes. Prev Med.

[20] Lagarde M, Haines A, Palmer N (2009). The impact of conditional cash transfers on health outcomes and use of health services in low and middle income countries. Cochrane Database Syst Rev.

[21] Gopalan SS, Mutasa R, Friedman J, Das A (2014). Health sector demand-side financial incentives in low- and middle-income countries: a systematic review on demand- and supply-side effects. Soc Sci Med.

[22] Brown DS, Finkelstein EA, Brown DR, Buchner DM, Johnson FR (2009). Estimating older adults' preferences for walking programs via conjoint analysis. Am J Prev Med.

[23] Finkelstein EA, Brown DS, Brown DR, Buchner DM (2008). A randomized study of financial incentives to increase physical activity among sedentary older adults. Prev Med.

[24] Fieno J, Leclerc-Madlala S (2014). The promise and limitations of cash transfer programs for HIV prevention. Afr J AIDS Res.

[25] Galarraga O, Sosa-Rubi SG, Infante C, Gertler PJ, Bertozzi SM (2014). Willingness-to-accept reductions in HIV risks: conditional economic incentives in Mexico. Eur J Health Econ.

[26] Operario D, Kuo C, Sosa-Rubi SG, Galarraga O (2013). Conditional economic incentives for reducing HIV risk behaviors: integration of psychology and behavioral economics. Health Psychol.

[27] de Walque D, Dow WH, Medlin C, Nathan R (2012). Stimulating demand for AIDS prevention - lessons from the RESPECT trial. In: policy research working paper.

[28] de Walque D, Dow WH, Nathan R, Abdul R, Abilahi F, Gong E (2012). Incentivising safe sex: a randomised trial of conditional cash transfers for HIV and sexually transmitted infection prevention in rural Tanzania. BMJ Open.

[29] Baird SJ, Garfein RS, McIntosh CT, Ozler B (2012). Effect of a cash transfer programme for schooling on prevalence of HIV and herpes simplex type 2 in Malawi: a cluster randomised trial. Lancet.

[30] Lee R, Cui RR, Muessig KE, Thirumurthy H, Tucker JD (2014). Incentivizing HIV/STI testing: a systematic review of the literature. AIDS Behav.

[31] Haukoos JS, Witt MD, Coil CJ, Lewis RJ (2005). The effect of financial incentives on adherence with outpatient human immunodeficiency virus testing referrals from the emergency department. Acad Emerg Med.

[32] Nglazi MD, van Schaik N, Kranzer K, Lawn SD, Wood R, Bekker LG (2012). An incentivized HIV counseling and testing program targeting hard-to-reach unemployed men in Cape Town, South Africa. J Acquir Immune Defic Syndr.

[33] Thornton R (2008). The demand for, and impact of learning HIV status. Am Econ Rev.

[34] Datta S, Mullainathan S (2014). Behavioral design: a new approach to development policy. Rev Income Wealth.

[35] 2012 Tanzania population and housing census [http://www.nbs.go.tz/sensa/index.html]

[36] Kapiga SH, Sam NE, Shao JF, Renjifo B, Masenga EJ, Kiwelu IE (2002). HIV-1 epidemic among female bar and hotel workers in northern Tanzania: risk factors and opportunities for prevention. J Acquir Immune Defic Syndr.

[37] Ao TT, Sam NE, Masenga EJ, Seage GR, Kapiga SH (2006). Human immunodeficiency virus type 1 among bar and hotel workers in northern Tanzania: the role of alcohol, sexual behavior, and herpes simplex virus type 2. Sex Transm Dis.

[38] Peaty D. Kilimanjaro tourism and what it means for local porters and for the local Enviroment. J Ritsumeikan Soc Sci and Humanities 2012;4:1–11

[39] Deane KD, Parkhurst JO, Johnston D (2010). Linking migration, mobility and HIV. Trop Med Int Health.

[40] Delany-Moretlwe S, Bello B, Kinross P, Oliff M, Chersich M, Kleinschmidt I (2013). HIV prevalence and risk in long-distance truck drivers in South Africa: a national cross-sectional survey. Int J STD AIDS.

[41] Smolak A (2014). A meta-analysis and systematic review of HIV risk behavior among fishermen. AIDS Care.

[42] Kiwanuka N, Ssetaala A, Mpendo J, Wambuzi M, Nanvubya A, Sigirenda S (2013). High HIV-1 prevalence, risk behaviours, and willingness to participate in HIV vaccine trials in fishing communities on Lake Victoria, Uganda. J Int AIDS Soc.

[43] Kwena ZA, Bukusi EA, Ng'ayo MO, Buffardi AL, Nguti R, Richardson B (2010). Prevalence and risk factors for sexually transmitted infections in a high-risk occupational group: the case of fishermen along Lake Victoria in Kisumu Kenya. Int J STD AIDS.

[44] Clift S, Anemona A, Watson-Jones D, Kanga Z, Ndeki L, Changalucha J (2003). Variations of HIV and STI prevalences within communities neighbouring new goldmines in Tanzania: importance for intervention design. Sex Transm Infect.

[45] Desmond N, Allen CF, Clift S, Justine B, Mzugu J, Plummer ML (2005). A typology of groups at risk of HIV/STI in a gold mining town in north-western Tanzania. Soc Sci Med.

[46] Heffron R, Chao A, Mwinga A, Sinyangwe S, Sinyama A, Ginwalla R (2011). High prevalent and incident HIV-1 and herpes simplex virus 2 infection among male migrant and non-migrant sugar farm workers in Zambia. Sex Transm Infect.

[47] Ostermann J, Whetten K, Reddy E, Pence B, Weinhold A, Itemba D (2014). Treatment retention and care transitions during and after the scale-up of HIV care and treatment in Northern Tanzania. AIDS Care.

[48] Ostermann J, Njau B, Brown DS, Muhlbacher A, Thielman N (2014). Heterogeneous HIV testing preferences in an urban setting in tanzania: results from a discrete choice experiment. PLoS One.

[49] Ostermann J, Njau B, Mtuy T, Brown D, Muehlbacher A. One size does not fit all: HIV testing preferences differ among high-risk groups in Northern Tanzania. AIDS care 2014, in press. 10.1080/09540121.2014.998612 PMC4336601 25616562

[50] World Health Organization. Module 7. The EPI coverage survey. In: Training for mid-level managers (MLM). Volume WHO/IVB/08.07 edn. Geneva, Switzerland; 2008.

[51] Kickbusch IS (2001). Health literacy: addressing the health and education divide. Health Promot Int.

[52] Morrill AC, Noland C (2006). Interpersonal issues surrounding HIV counseling and testing, and the phenomenon of "testing by proxy". J Health Commun.

[53] DiCarlo AL, Mantell JE, Remien RH, Zerbe A, Morris D, Pitt B (2014). 'Men usually say that HIV testing is for women': gender dynamics and perceptions of HIV testing in Lesotho. Cult Health Sex.

[54] Singh K, Luseno W, Haney E (2013). Gender equality and education: Increasing the uptake of HIV testing among married women in Kenya, Zambia and Zimbabwe. AIDS Care.

[55] Sia D, Onadja Y, Nandi A, Foro A, Brewer T (2013). What lies behind gender inequalities in HIV/AIDS in sub-Saharan African countries: evidence from Kenya, Lesotho and Tanzania. Health Policy Plan.

[56] Landman KZ, Ostermann J, Crump JA, Mgonja A, Mayhood MK, Itemba DK (2008). Gender differences in the risk of HIV infection among persons reporting abstinence, monogamy, and multiple sexual partners in northern Tanzania. PLoS One.

[57] Weesie J (1999). sg121: Seemingly unrelated estimation and the cluster-adjusted sandwich estimator. Stata Tech Bull.

[58] Lopez-Feldman A. Introduction to Contingent Valuation using Stata. In*.* Munich Personal RePEc Archive: Centro de Investigacion y Docencia Economicas (Cide); 2012.

[59] Hanemann M, Loomis J, Kanninen B (1991). Statistical efficiency of double-bounded dichotomous choice contingent valuation. Am J Agricul Econ.

[60] TACAIDS, Zanzibar AIDS Commission, National Bureau of Statistics, Office of the Chief Government Statistician, ICF International (2013). HIV/AIDS and malaria indicator survey 2011–12.

[61] Kranzer K, Govindasamy D, Ford N, Johnston V, Lawn SD (2012). Quantifying and addressing losses along the continuum of care for people living with HIV infection in sub-Saharan Africa: a systematic review. J Int AIDS Soc.

[62] Global AIDS Response Country Progress Report [http://www.unaids.org/sites/default/files/en/dataanalysis/knowyourresponse/countryprogressreports/2014countries/TZA_narrative_report_2014.pdf]

[63] Waters RC, Ostermann J, Reeves TD, Masnick MF, Thielman NM, Bartlett JA (2011). A cost-effectiveness analysis of alternative HIV re-testing strategies in sub-Saharan Africa. J Acquir Immune Defic Syndr.

[64] GDP per capita (current US$) [http://data.worldbank.org/indicator/NY.GDP.PCAP.CD]

[65] Mukolo A, Villegas R, Aliyu M, Wallston KA (2013). Predictors of late presentation for HIV diagnosis: a literature review and suggested way forward. AIDS Behav.

[66] Mayhood MK, Afwamba IA, Odhiambo CO, Ndanu E, Thielman NM, Morrissey AB (2008). Validation, performance under field conditions, and cost-effectiveness of Capillus HIV-1/HIV-2 and determine HIV-1/2 rapid human immunodeficiency virus antibody assays using sequential and parallel testing algorithms in Tanzania. J Clin Microbiol.

[67] Dahl V, Mellhammar L, Bajunirwe F, Bjorkman P (2008). Acceptance of HIV testing among women attending antenatal care in south-western Uganda: risk factors and reasons for test refusal. AIDS Care.

[68] Angotti N, Bula A, Gaydosh L, Kimchi EZ, Thornton RL, Yeatman SE (2009). Increasing the acceptability of HIV counseling and testing with three C's: convenience, confidentiality and credibility. Soc Sci Med.

[69] Musheke M, Ntalasha H, Gari S, McKenzie O, Bond V, Martin-Hilber A (2013). A systematic review of qualitative findings on factors enabling and deterring uptake of HIV testing in Sub-Saharan Africa. BMC Public Health.

[70] Lagarde M, Haines A, Palmer N (2007). Conditional cash transfers for improving uptake of health interventions in low- and middle-income countries: a systematic review. JAMA.

[71] Pettifor A, MacPhail C, Nguyen N, Rosenberg M (2012). Can money prevent the spread of HIV? A review of cash payments for HIV prevention. AIDS Behav.

[72] Hensen B, Baggaley R, Wong VJ, Grabbe KL, Shaffer N, Lo Y-RJ, et al. Universal voluntary HIV testing in antenatal care settings: a review of the contribution of provider-initiated testing & counselling. Tropical medicine & international health: TM & IH 2012;17(1):59–70. 10.1111/j.1365-3156.2011.02893.x 22032300

[73] Dow WH, White JS. Incentivizing use of health care. In. United Nations Population Division Publications. 2013.

[74] London AJ, Borasky Jr DA, Bhan A. For the Ethics Working Group of the HIVPTN: Improving Ethical Review of Research Involving Incentives for Health Promotion. PLoS Med 2012;9(3):e1001193. 10.1371/journal.pmed.1001193 PMC3313933 22479154

[75] Ensor T, Cooper S (2004). Overcoming barriers to health service access: influencing the demand side. Health Policy Plan.

[76] Stecklov G, Winters P, Todd J, Regalia F (2007). Unintended effects of poverty programmes on childbearing in less developed countries: experimental evidence from Latin America. Popul Stud (Camb).

[77] Kohler HP, Thornton R (2012). Conditional Cash Transfers and HIV/AIDS Prevention: Unconditionally Promising?. World Bank Econ Rev.

[78] Deci EL, Koestner R, Ryan RM (1999). A meta-analytic review of experiments examining the effects of extrinsic rewards on intrinsic motivation. Psychol Bull.

[79] Boyle K, Bishop R, Welsh M (1985). Starting Point Bias in Contingent Valuation Bidding Games. Land Econ.

[80] Green D, Jacowitz KE, Kahneman D, McFadden D (1998). Referendum contingent valuation, anchoring, and willingness to pay for public goods. Resour Energ Econ.

[81] Niza C, Rudisill C, Dolan P (2014). Vouchers versus Lotteries: What Works Best in Promoting Chlamydia Screening? A Cluster Randomized Controlled Trial. Appl Econ Perspect Policy.

[82] Cummings RG, Harrison GW, Rutström EE (1995). Homegrown values and hypothetical surveys: is the dichotomous choice approach incentivecompatible?. Am Econ Rev.

[83] Cummings RG, Elliot S, Harrison GW, Murphy J (1997). Are hypothetical referenda incentive compatible?. J Political Econ.

